# The Cytotoxicity of RNase-Derived Peptides

**DOI:** 10.3390/biom11010016

**Published:** 2020-12-26

**Authors:** Vera Ulyanova, Elena Dudkina, Alsu Nadyrova, Vladimir Kalashnikov, Yulia Surchenko, Olga Ilinskaya

**Affiliations:** Department of Microbiology, Institute of Fundamental Medicine and Biology, Kazan Federal University, 420008 Kazan, Russia; ulyanova.vera@gmail.com (V.U.); alsu.nadyrova@yandex.ru (A.N.); icebuldogq@gmail.com (V.K.); sokurenko.yulia@gmail.com (Y.S.); ilinskaya_kfu@mail.ru (O.I.)

**Keywords:** anticancer peptides, ribonuclease (RNase), binase, cytotoxicity, EGF, epidermal growth factor (EGFR)

## Abstract

Bacterial ribonuclease binase exhibits a cytotoxic effect on tumor cells possessing certain oncogenes. The aim of this study was to identify the structural parts of the binase molecule that exert cytotoxicity. Out of five designed peptides, the peptides representing the binase regions 21–50 and 74–94 have the highest cytotoxic potential toward human cervical HeLa and breast BT-20 and MCF-7 cancer cells. The peptides B21–50 and B74–94 were not able to enter human lung adenocarcinoma A549 cells, unlike BT-20 cells, explaining their failure to inhibit A549 cell proliferation. The peptide B74–94 shares similarities with epidermal growth factor (EGF), suggesting the peptide’s specificity for EGF receptor overexpressed in BT-20 cells. Thus, the binase-derived peptides have the potential of being further developed as tumor-targeting peptides.

## 1. Introduction

The main disadvantages of traditional anticancer therapy are the lack of selectivity, arising drug resistance, and side effects of chemotherapeutic agents. For this reason, anticancer peptides (ACPs) are considered as promising tools in antitumor treatment [1]. ACPs have several advantages over other chemotherapeutics such as high specificity, short time-frame of interaction, good tumor penetration, good solubility, high affinity, and low toxicity that decrease the probability of resistance emergence and reduce the side effects of ACPs [2,3,4]. ACPs generally consist of 5 to 50 amino acid residues folded into helical structures; however, other conformations are encountered as well [2,5,6]. The anticancer properties of ACPs are attributed to their amphipathicity, moderate overall hydrophobicity, and positive net charge [7,8]. Membranolytic, apoptosis-inducing, necrosis-triggering, immune-stimulating, angiogenesis-inhibiting modes of ACPs action are reported [6]. Cell membrane charge and composition is thought to account for the selective toxicity of many ACPs toward cancer cells. However, the mechanisms of ACPs activity and selectivity toward cancer cells still are not fully understood.

Among potential therapeutics, special attention is being paid to compounds affecting the specific RNAs of tumor cells. Ribonucleases (RNases) of animal, fungal, and bacterial origin have shown promise as a tool for the development of novel anticancer drugs [9,10,11,12,13]. It has been proven that the antitumor potential of RNases is mediated by their catalytic activity [14,15], the charge of the molecule [10,16], protein structural organization [17,18], and interaction with cellular components [19,20]. It has been shown that positively charged dimeric RNase from *Bacillus pumilus* (binase) [17,21] selectively inhibits tumor growth via the induction of apoptosis in cancer cells [14,22,23], but molecular mechanisms underlying this biologic effect require a detailed explanation. Earlier, it was believed that the cytotoxicity of RNases is predominantly due to their catalytic activity toward available RNA; at the same time, RNA species other than rRNA and/or tRNA were supposed to be targeted as well [24]. It has been shown that the inhibition of tumor and metastasis growth by pancreatic RNase A is accompanied by the global alteration of miRNA profiles in the blood and tumor tissue [14]. However, the alteration of total RNA level induced by binase is not fatal for cell viability [25]. Except for the reaction of bacterial and eukaryotic RNases with their substrates, practically nothing is known about the interaction of these enzymes with other cellular components. For binase, the selective cytotoxicity toward cancer cell lines expressing certain oncogenes, namely ras, kit, acute myelogenous leukemia 1 AML1) transcription factor and the eight-twenty one (ETO) corepressor, and FMS-like tyrosine kinase 3 (FLT3), has been previously demonstrated [15,20,22,26,27]. It is still unclear what exactly leads to the upregulation of carbohydrate metabolism, inositol phosphate cascade, oxidative phosphorylation, cellular transport and localization, re-arrangement of cell adhesion, cell cycle control, apoptosis, and transcription in tumor cells following RNase treatment [28,29]. Binase has been shown to induce tumor cell death via inhibition of the mitogen-activated protein kinase (MAPK)/extracellular signal regulated kinase (ERK) signaling pathway through direct interaction with Kirsten rat sarcoma viral oncogene homolog protein (KRAS) [20]. Thus, the antitumor effect induced by RNases is associated with the different molecular events that these enzymes trigger in cancer cells.

Here, we have investigated the ability of binase-derived peptides to induce similar cytotoxic effect as the full-length protein for their further development into ACPs. We have proposed that peptides derived from binase will be more specific and efficient in the inhibition of tumor cell proliferation when compared to the entire protein. In addition, they should have less side effects and avoid undesirable immune responses in patients [2,3,4]. To identify the binase regions contributing to its cytotoxic potential, we have designed five peptides based on the binase three-dimensional structure and assessed their cytotoxicity toward different cancer cell lines.

## 2. Materials and Methods

### 2.1. Binase and Binase-Derived Peptides

The authentic ribonuclease from *B. pumilus* (12.3 kDa, pI 9.5) was purified as described earlier [30]. Binase-derived peptides were synthesized by GenScript Corporation (Piscataway, NJ, USA). The purity of the peptides was at least 90%. Binase-derived peptides B21–50 and B74–94 possessing 6x-His affinity tag at the N-terminus were synthesized by Elabscience Biotechnology (Dallas, TX, USA).

### 2.2. Cell Cultures

The human alveolar adenocarcinoma cell line (A549), two breast cancer cell lines (MCF-7 and BT-20), and cervical cancer cell line (HeLa) were obtained from American Type Culture Association (Rockville, MD, USA). Cells were grown at 37 °C in humidified 5% CO_2_ atmosphere using RPMI 1640 medium for A549 cells and Dulbecco’s modified Eagle’s medium for MCF-7, BT-20, and HeLa cells supplemented with 10% fetal bovine serum (HyClone), 2 mM glutamine, and antibiotics (penicillin and streptomycin, 100 U/mL each).

### 2.3. MTT (3-(4,5-dimethylthiazol-2-yl)-2,5-diphenyltetrazolium bromide) Assay

The viability of untreated control cells and cells treated with binase and binase-derived peptides at different concentrations (5, 50, and 125 μM) was determined according to the mitochondrial nicotinamide adenine dinucleotide phosphate NAD(P)H-dependent cellular oxidoreductases activity tested by the standard procedure based on the reduction of the tetrazolium dye MTT) to its insoluble product formazan, which has a purple color. Cells at the initial concentration of 104 per well were grown in a 96-well plate (CELLTREAT Scientific Products, Shirley, MA, USA); then, the culture fluid was discarded and fresh medium with peptides was added. After 48 h, formazan absorption was measured at 570 nm (xMark, Bio-Rad, Hercules, CA, USA). The amount of formazan produced is proportional to the number of viable cells. The viability of untreated cells was taken as 100%.

To examine the peptide effect on EGFR signaling, the EGFR-specific monoclonal antibody cetuximab (Merck, KGaA, Darmstadt, Germany) was added at the concentration of 10 μg/mL to cells grown for 24 h in 96-well plates. After 48 h of exposure to inhibitor, half the wells were treated with the B74–94 peptide at 50 μM concentration, and the cells were allowed to grow for another 48 h. Afterwards, cell proliferation was assessed by the MTT assay.

### 2.4. Peptide Characterization and Modeling

The ExPASy ProtParam tool (http://www.expasy.ch/tools/protparam.html) and the Prot pi Protein Tool (https://www.protpi.ch/Calculator/ProteinTool) were used to calculate the physical and chemical parameters of the peptides, namely length, molecular weight (MW), pI, net charge, instability index, aliphatic index, and grand average of hydropathicity (GRAVY). The peptides were predicted to be putative ACPs based on amino acid composition, conserved features, and physicochemical properties by AntiCP [31]. The hydrophobicity, amphiphaticity, total hydrophobic ratio, and Boman index were computed by AntiCP and APD3 [32]. These peptides were checked by ToxinPred for the prediction of toxic peptides [33] and by CellPPD for the prediction of cell penetrating properties [34]. Peptides were modeled by the de novo peptide structure prediction tool PEP-FOLD3 using 200 simulation runs to sample the conformations [35]. Models were sorted using the sOPEP energy value, and the best ranked peptide models were selected. Jmol: an open-source Java viewer for chemical structures in 3D (http://www.jmol.org/) was used to visualize 3D structures. Pairwise and multiple comparison of protein structures was performed using FATCAT and POSA [36]. Docking of binase-derived peptides was performed on ClusPro Server using Receptor-Ligand mode [37] and by the GRAMM-X protein–protein docking server [38]. For local refinement of the of peptide–protein complex structures, the Rosetta FlexPepDock server (http://flexpepdock.furmanlab.cs.huji.ac.il/) was used. The binding pose with the lowest interaction score was analyzed.

### 2.5. Immunofluorescence Microscopy

A549 cells (25,000 cells/well) and BT-20 cells (150,000 cells/well) were seeded on 4-well chamber slides and incubated for 24 h in 800 µL of 10% fetal bovine serum containing RPMI or DMEM, respectively. The medium was replaced with the fresh one, and the His-tagged B21–50 and B74–94 peptides or tagless binase were applied to each well at the final concentration of 50 µM. After 30 min of incubation, the medium was removed, and the cells were washed three times with ice-cold phosphate-buffered saline (PBS). Then, cells were fixed in a 4% paraformaldehyde solution for 15 min and permeabilized in 0.1% Triton-X100 solution in PBS for 10 min. Binase-treated cells were incubated overnight with anti-binase antibodies (1:500) at 4 °C [39]. Thereafter, the cells were washed three times in PBS containing 0.1% Tween for 10 min each and then were incubated with fluorescein isothiocyanate (FITC)-conjugated mouse anti-rabbit immunoglobulin G (IgG) (H+L) cross-adsorbed secondary antibody (Thermo Fisher Scientific, Inc., Waltham, MA, USA Cat.# 31584) at the concentration of 2 µg/mL in PBS containing 1% bovine serum albumin at room temperature for 45 min in the dark. Non-specific binding of the secondary antibodies was evaluated at the same experiment but without treatment with primary antibodies. Peptide-treated cells were stained with anti-His-probe Alexa Fluor 647-conjugated mouse monoclonal antibodies (sc-53073, Santa Cruz Biotechnology, Inc., Dallas, TX, USA) at a dilution of 1:50 and incubated for 2 h at room temperature in the dark. Nuclear DNA was labeled with 4′,6-diamidino-2-phenylindole (DAPI) for 15 min at 37 °C. Confocal laser scanning microscope observations were conducted using an LSM 700 instrument (Carl Zeiss AG, Jena, Germany) with a Plan-Apochromat 63×/1.4 objective (Carl Zeiss) at the 405 nm excitation wavelength of laser for DAPI, 647 nm laser for Alexa Fluor 647, and 488 nm laser for FITC.

### 2.6. Statistical Analysis

All experiments were performed in triplicate. All data are presented as the mean ± standard deviation of the mean (SD). Multiple groups were compared by two-way analysis of variance (ANOVA) with Dunnett’s multiple comparison test. Statistical tests and graphical data presentation were performed using GraphPad Prism 8 software (GraphPad Software, San Diego, CA, USA).

## 3. Results and Discussion

### 3.1. Characterization of Anticancer Potential of Binase-Derived Peptides

**Figure 1:**
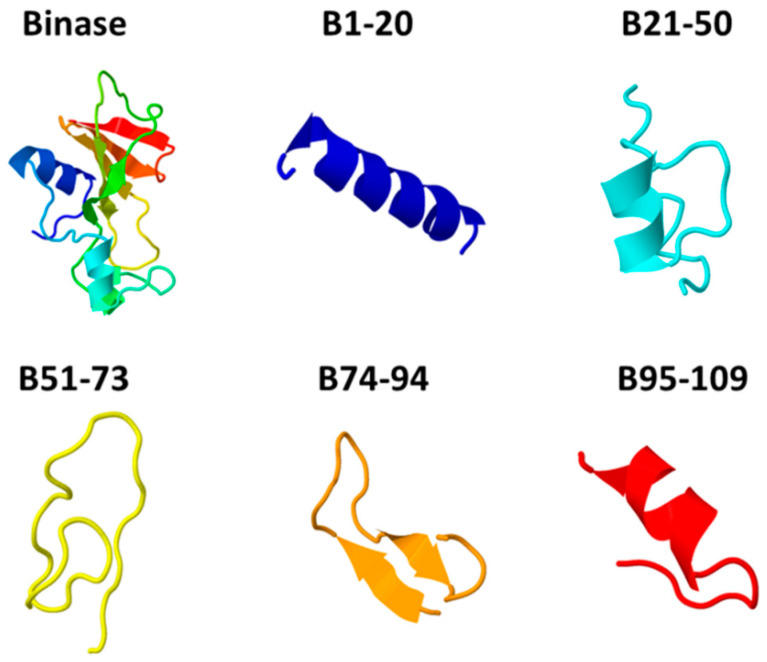
The models of three-dimensional structure of binase-derived peptides in comparison to the binase structure in solution (PDB 1BUJ). The numbers correspond to the numbering of amino acid residues in a binase primary sequence. The color of the models matches the rainbow coloring of the binase spatial structure.

To determine the binase regions contributing to its cytotoxicity, we have virtually cut the binase into several parts (Figure 1): 1–20 (the first α-helix), 21–50 (the second and the third α-helices, two-stranded parallel β-sheet 1, flexible loop 1), 51–73 (strands 1 and 2 of the antiparallel β-sheet 2, flexible loop 2), 74–94 (strand 3 of the antiparallel β-sheet 2, flexible loop 3), and 95–109 (strands 4 and 5 of the antiparallel β-sheet 2) [40]. Peptides corresponding to each of these regions have been chemically synthesized. Three-dimensional models of all peptides, except for the B95–109, have been predicted to have nearly the same topology as in the whole binase molecule (Figure 1).

According to the calculated physicochemical properties, the majority of the peptides, except for B74–94, are basic molecules such as the entire binase (Table 1). The peptide B95–109 has the most positive net charge but are likely to be less stable in vitro than others. The hydrophobicity of the peptides decreases in the rows B1–20 ≥ B21–50 > B74–94 > B95–109 ≥ B51–73 (Table 2). The highest amphipathicity is attributed to B95–109. The peptides B51–73 and B95–109 have the Boman index higher than two, which gives an overall estimate of their high potential to bind other proteins [31].

ACPs share similar properties with antimicrobial peptides; most of them are positively charged, amphiphilic either α-helical or β-sheet peptide molecules [4]. Recent studies aimed at determining of physicochemical properties that are responsible for antitumor activity of peptides have shown that the most important role in antitumor potential is played by the amphipathicity, hydrophobicity, and overall positive charge of peptide molecules [8,41,42]. The equilibrium between these parameters is more significant than exact values [43]. Moreover, the structural organization and the susceptibility of peptides to proteolysis contribute to their cytotoxicity [4]. Most ACPs do not have a well-defined structure when free in solution, but they adopt an α-helix or β-sheet structure upon electrostatic interactions with membranes [44].

Thus, based on the structural and physicochemical properties, the peptides B1–20, B21–50, and B95–109 represent the best candidates for ACPs. However, the antiCP server, which takes into account different parameters of peptides cumulatively, has predicted the peptides B21–50 and B74–94 to be potential ACPs (Table 2). At the same time, none of the peptides have been predicted to have toxic and membrane-damaging properties.

### 3.2. Cytotoxic Effects of Binase-Derived Peptides toward HeLa, A549, BT-20, and MCF-7 Cells

**Figure 2:**
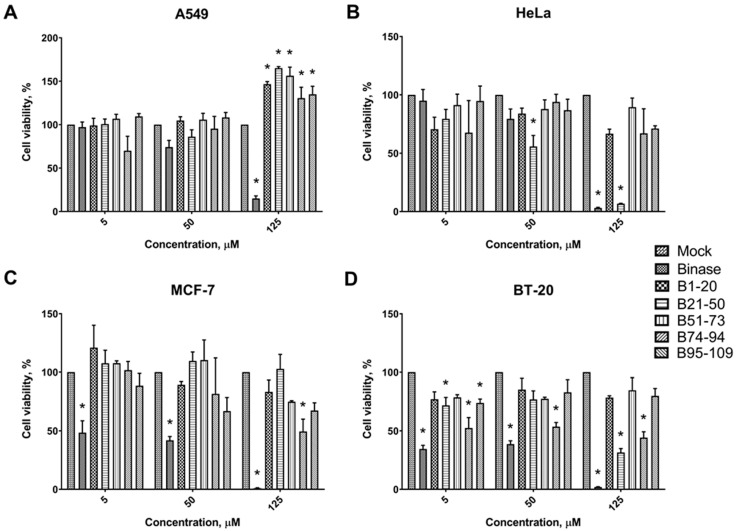
The effect of binase and binase-derived peptides on the cell viability of the A549 cells (**A**), the HeLa cells (**B**), the MCF-7 cells (**C**), and the BT-20 cells (**D**) as measured via the MTT (3-(4,5-dimethylthiazol-2-yl)-2,5-diphenyltetrazolium bromide) assay. Cells were incubated with the indicated concentrations of the compounds for 48 h. The viability of untreated cells was taken for 100%. Data are representative of three independent experiments; * *p* < 0.0001 as determined by two-way ANOVA with Dunnett’s post-hoc test for multiple comparisons.

To elucidate the cytotoxicity of binase-derived peptides, we have performed MTT assay based on the reduction of tetrazolium dye to formazan by mitochondrial dehydrogenases, whose activity correlates to respiration and allows evaluating cell viability. The viability of A549 cells treated with 5, 50, and 125 µM of the full-length binase has decreased by 3%, 26%, and 85% respectively after 48 h of incubation (Figure 2), which corresponds to previously obtained data [45]. All the peptides at the concentrations 5 and 50 µM have not affected A549 cell viability; it has remained at the level of untreated cells (Figure 2A). Increased concentrations (125 µM) of the peptides have a stimulating effect: cell proliferation has risen by 30–65%. So, the binase-derived peptides have no cytotoxic effect on A549 cells at all tested concentrations.

The antiproliferative effect has been observed in the HeLa cell line after treatment by increasing concentrations of binase-derived peptides, in particular the peptide B21–50 (Figure 2B). When 50 and 125 µM of the B21–50 peptide have been applied, the viability of HeLa cells after 48 h incubation has been statistically significantly lowered by 45% and 93% respectively, whereas binase itself at the highest concentration has decreased the viability by 97%.

In the MCF-7 cell line, the percentage of viable cells after binase treatment at 5, 50, and 125 µM has decreased by 52%, 58%, and 99%, respectively (Figure 2C). The B74–94 was the only binase-derived peptide that has shown cytotoxicity toward MCF-7 cells, inhibiting cell proliferation two-fold at 125 µM concentration.

In our experiment, BT-20 cells were the most sensitive cell line to binase and its peptides (Figure 2D). The viability of BT-20 cells treated with 5, 50, and 125 µM of the full-length binase has decreased by 65%, 61%, and 98%, respectively, after 48 h of cell treatment as compared to non-treated cells. The similar cytotoxic effect has been demonstrated by binase-derived peptides B21–50 and B74–94. The peptide B21–50 at the same concentrations has reduced cell viability by 28%, 23% and 68%, respectively. The administration of the peptide B74–94 has significantly inhibited the proliferation of BT-20 cells approximately by 50% at either concentration. The peptides B1–20, B51–73, and B95–109 were also capable of decreasing cell viability but only by approximately 15–25%.

Thus, based on MTT assay, it has been found that peptides from binase regions 21–50 and 74–94 have the highest cytotoxic potential as compared with the other binase-derived peptides. Being cytotoxic, these two peptides have completely different physicochemical properties. The peptide B21–50 is a cationic and helical molecule, while the peptide B74–94 has a negative net charge and a β-sheet conformation (Figure 1, Table 1 and Table 2). The peptide B21–50 is slightly more amphipathic and hydrophobic than the peptide B74–94; as predicted, it has four hydrophobic amino acid residues exposed on the same surface, while the peptide B74–94 has six residues, which is regarded as a positive aspect of ACPs. Therefore, the experimental results have corroborated the computational prediction of anticancer properties of designed peptides and have underlined the importance of balance between molecule characteristics.

**Figure 3:**
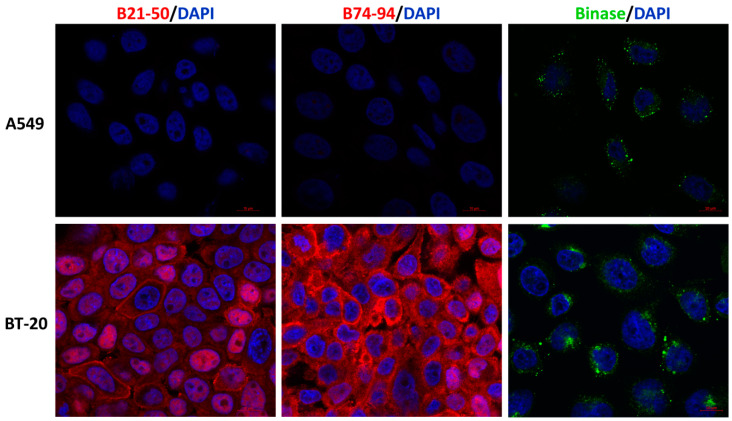
Confocal laser scanning images of A549 and BT-20 cells treated with His-tagged B21–50 and B74–94 peptides or binase. Peptides (red), binase (green), and nuclei (blue) were stained using anti-His-probe Alexa Fluor 647-conjugated mouse monoclonal antibodies, FITC-conjugated mouse anti-rabbit IgG (H+L) cross-adsorbed secondary antibodies and DAPI, respectively. Scale bars represent 10 µm.

Obviously, the question arises of why the peptides have manifested toxicity toward BT-20, MCF-7, and HeLa cells in contrast to A549 cells. This can be explained by inability of peptides to enter cells or by their proteolytic degradation. The poor cell permeability is one of the limitations of therapeutic peptides. Cell-penetrating ability is considered to be important for selective cytotoxicity. It depends on the physicochemical properties, length, concentration, and charge of peptide molecules [46]. Peptides can cross the cell membrane through energy-independent direct penetration or energy-dependent endocytosis mechanisms [47]. Several models have been reported for peptides penetration, but the exact mechanism is still obscure [48]. We have determined the ability of B21–50 and B74–94 peptides to enter BT-20 and A549 cells as the most sensitive and insensitive cells to their action, respectively. Both peptides (50 µM) have been detected inside BT-20 cells by confocal laser scanning microscopy after 30 min incubation with cells; they were distributed throughout the cell, in cytoplasm and nuclei (Figure 3). At the same time, both peptides have not been visualized in A549 cells, potentially explaining the absence of their cytotoxicity toward this cell line. Distinctively, binase has been detected inside both BT-20 and A549 cells (Figure 3).

The possibility of being degraded by multiple serum and tissue proteases with differing specificity limits the systemic application of therapeutic peptides. Host proteases play an important role in determining the cell or tissue tropism of different viruses [48]. Lung epithelial cells express matrix metalloproteinases (MMPs) and type II transmembrane serine proteases (TTSPs); among them transmembrane serine protease 4 (TMPRSS4) and 15 (TMPRSS15) are found in A549 cells though at a low expression level [49,50,51]. Both proteases regulate physiological processes as well as tumor development and progression notably in lung, pancreatic, thyroid, colon, and gastric cancers [52]. They cleave and activate growth factors and signaling receptors as well as facilitate cell migration, invasion, and angiogenesis [52]. TMPRSS4 and TMPRSS15 are not found in breast cancer cells [53,54]; however, these cells produce other proteases, namely TMPRSS13 and MMP1, 13, 14, and 23 [55]. In our work, we have not detected any difference in proteolytic activity between the culture media of A549, BT-20, HeLa, and MCF-7 cell lines during 72 h of incubation (data not shown). Therefore, the absence of the B21–50 and B74–94 peptides in A549 cells is not connected to the extracellular degradation of the peptides. For A549 cells, the multidrug-resistance to chemotherapy mediated by the process of autophagy is reported [56,57]. It has been shown that autophagy depends on the activity of cellular proteases and results in the decreasing of apoptotic potential of cancer cells and enhancing of their survival [58]. The increased autophagy during chemotherapy has also been recognized in a luminal type of breast cancer (MCF-7 cell line) but not in the triple-negative one [59]. In the triple-negative breast cancer BT-20 cell line, inhibition of autophagy is linked to the overexpression of the epidermal growth factor receptor (EGFR) [60]. So, it cannot be ruled out that the increased level of protease activity inside the A549 and MCF-7 cells leads to the digestion of peptides into the individual amino acids, which are used by cells as an additional nutrition source causing growth stimulation.

The specificity of the peptides action toward certain cell lines can be coupled with certain cell mutations or differences in cell surface composition. For instance, A549 cells differ from other cell lines by the strong permanent activation of MAPK/ERK signaling pathway due to the G12S mutation in KRAS protein. It can be supposed that binase-derived peptides are not powerful enough to inhibit the constantly active signaling pathway. In addition to pore-forming and cell-permeable peptides, a group of tumor-targeting peptides is distinguished among ACPs [61]. These peptides target certain markers expressed on the tumor cell membrane, such as integrins, CD13, and other receptors, and they can be internalized into the cell through receptor-mediated endocytosis [62]. According to their biological targets, therapeutic peptides can be divided into three groups influencing signal transduction, cell cycle regulation, and cell death pathways [63]. Since binase-derived peptides has selectively penetrated and inhibited the proliferation of different cancer cell lines, we have proposed that their cytotoxicity is due to the interaction with specific receptors or intracellular targets. Among four cancer cell lines, the most sensitive to binase-derived peptides was BT-20. This cell line is characterized as a triple negative subtype of breast cancers that do not express progesterone receptors, estrogen receptors, and human epidermal growth factor receptor 2 [64]. In BT-20 cells, the epidermal growth factor receptor (EGFR) is overexpressed, and its targeting is suggested as one of the strategies in therapy of triple-negative breast cancer [65].

### 3.3. Assessment of Putative Interaction between EGFR and Binase-Derived Peptides

Receptor tyrosine kinase EGFR (ErbB1, HER1) belongs to a four-member ErbB family, which plays a pivotal role in signal transduction that controls cell division and survival. Therefore, alterations in the functioning of ErbB proteins correlate with the development and progression of numerous human cancers, including lung, breast, ovarian, and other types [66]. Seven polypeptide growth factors were shown to bind EGFR, i.e., epidermal growth factor (EGF), amphiregulin (AREG), transforming growth factor (TGF)-and epigen, which bind EGFR specifically, betacellulin (BTC), heparin-binding EGF (HB-EGF), and epiregulin (EPR), which are capable of binding both EGFR and ErbB4 [66]. In the absence of ligand, EGFR exists as an inactive monomer. Upon ligand binding, EGFR dimerizes into its active form followed by autophosphorylation, endocytosis and activation of downstream proteins that enhance cell proliferation, invasion, and metastasis and inhibit apoptosis.

**Figure 4:**
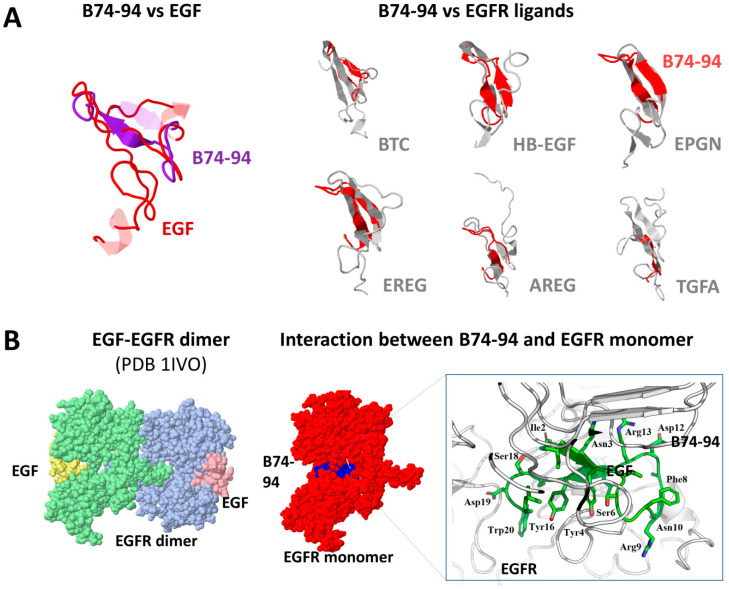
Molecular modeling of the interaction between the peptide B74–94 and EGFR. (**A**) Superposition of B74–94 peptide (purple) with EGF (red, PDB 2KV4) and other EGFR ligands. (**B**) The model of EGFR interaction with EGF (PDB 1IVO) and B74–94 peptide. The detailed view of EGFR and B74–94 interface is zoomed in.

To test the possibility of whether EGFR could be a potential target for binase-derived peptides, we have initially compared them with the known EGFR ligands. EGFR ligands have distinct physicochemical properties such as the binase-derived peptides (Table 1). The most similar to EGF is the peptide B74–94; both proteins are negatively charged and have similar hydropathy indexes. The highest similarity in the three-dimensional structure to EGF has been found in the peptides B74–94 (21 equivalent positions out of 21 amino acid residues of the peptide with an RMSD of 2.56), B21–50 (22 equivalent positions out of 30 amino acid residues of the peptide with an RMSD of 3.55), and B51–73 (17 equivalent positions out of 23 amino acid residues of the peptide with an RMSD of 3.29). However, only the structure of the peptide B74–94 has overlaid completely (all 21 amino acid residues of the peptide) structures of different EGFR ligands, suggesting the putative specificity of the peptide for EGFR (Figure 4A). The region of structural similarity is represented by the A (residues 6–19) and B (residues 20–31) loops of EGF, which constitute the hydrophobic surface on the protein required for the receptor recognition and binding. Since all the EGFR ligands have similar structures of their B-loop fragments, it is the primary structure that mainly accounts for differences in affinity of EGFR ligands to the receptor [67]. These ligands elicit distinct conformations in EGFR and thereby different cellular responses [68].

The modeling of interaction between the peptide B74–94 and EGFR has shown that the peptide can bind EGFR at the same region as EGF but at a slightly different site (Figure 4B). EGF, similar to other EGFR ligands, binds the receptor at the beta sheets of domains I (residues 1–165) and III (residues 310–481). In particular, the B-loop of EGF binds to domain I of EGFR via hydrophobic interactions, while the A and C loops bind with domain III of EGFR through hydrophobic and electrostatic bonds. The B74–94 peptide also interacts with both EGFR domains using hydrophobic and electrostatic forces (the total Rosetta energy score of the complex is -41.259); however, the interaction occurs deeper in the ligand-binding pocket due to the smaller size of the peptide (Figure 4B). Thus, the Tyr4, Phe8, Tyr16, and Tyr 20 of the B74–94 peptide form hydrophobic bonds with Ile318 and Phe357 of the domain III of EGFR protein, while Arg9 and Asp19 of the peptide interact electrostatically with Asp323 and Arg285 of the receptor. The residues Ile2, Asn3, Ser6, Asp12, and Leu21 of the peptide B74–94 are involved in interaction with domain I (Gln8, Asn12, Leu38, ASn86, and Met87) of EGFR. Therefore, the peptide B74–94 potentially could interact with the EGF receptor, disturbing its activity.

**Figure 5:**
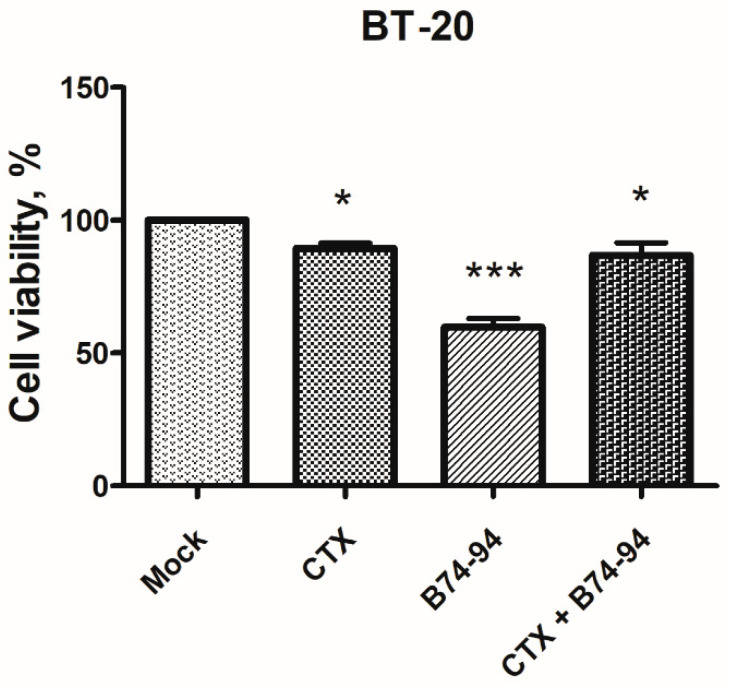
The effect of EGFR inhibition by cetuximab on cytotoxicity of the B74–94 peptide toward BT-20 cells as measured via the MTT assay. The viability of untreated cells was taken for 100%. Data are representative of three independent experiments; * *p* < 0.05, *** *p* < 0.001 as determined by two-way ANOVA with Dunnett’s post-hoc test for multiple comparisons.

To evaluate the possibility of the B74–94 peptide to interact with EGFR, we have performed the MTT assay with cetuximab, a monoclonal antibody that inhibits ligand binding upon interaction with the EGFR. We have found that preliminary cell treatment with 10 μg/mL cetuximab leads to a decrease of the B74–94 cytotoxicity by 28% (Figure 5). Probably, the cetuximab-mediated EGFR internalization affects the cytotoxicity of the B74–94 peptide by partial elimination of the peptide target from the cell surface. In addition to tyrosine kinase inhibitors and monoclonal antibodies, EGF antagonists are regarded as possible tools for inhibition of EGFR activity [69]. Several tumor-targeting peptides have been developed to target EGFR based on the structure of the natural EGF ligand [70,71]. They disrupt EGFR signaling by directly preventing ligand binding. Therefore, it is attractive to speculate that the cytotoxicity of the binase-derived peptide B74–94, which shares similar features with EGF, could be mediated by its interaction with EGFR; however, other targets cannot be ruled out.

## 4. Conclusions

The discovery of novel therapeutic peptides contributes to the development of potent anticancer therapy. Pharmaceutical compounds based on these small molecules are already approved by the United States Food and Drug Administration FDA and used for the illness treatment. In this work, we have characterized novel peptides derived from binase, an antitumor RNase of bacterial origin. Among five binase-derived peptides, the peptides from the regions 21–50 and 74–94 of binase molecule have exhibited high cytotoxic activity toward breast and cervical cancer cell lines. The peptides B21–50 and B74–94 have been computed to have a different structure and molecule net charge but similar stability and hydrophobicity. Their cytotoxicity is likely to be mediated by the balance of these properties as well as by the high cell-penetrating ability. The selectivity of the cytotoxic peptides toward distinct cancer cell lines indicates the presence of certain molecular targets, the blocking of which by the peptides leads to cancer cell death. For the peptide B74–94, such a target can be represented by the epidermal growth factor receptor amplified in the triple-negative cancer cell line BT-20 and overexpressed in several types of cancer. The peptide’s specificity for EGFR is predicted by its structural similarity with known EGFR ligands. Moreover, the EGFR blocking by the cetuximab has led to decrease of B74–94 cytotoxicity, indirectly indicating the targeting of EGFR. Although further study of direct protein–protein interactions is required, the high sensitivity of BT-20 cells to the binase-derived peptides can be linked to the overexpressed EGFR, since the prevention of EGFR activation can happen not only by blocking the growth factor binding sites on the receptor but also by stabilizing the receptor in the conformation that cannot bind growth factor with high affinity or by occluding the dimerization interface. However, other targets cannot be ruled out as well. Thus, the obtained results provide a clue for further development of certain binase-derived peptides as selective antitumor agents.

## Figures and Tables

**Table 1:** The physical and chemical parameters of the binase-derived peptides (A) and epidermal growth factor receptor (EGFR) ligands (B) calculated from their amino acid sequences.

	Peptide	MW, kDa	L ^1^, aa	Content of aa ^2^, %				pI	NCh at pH 7 ^3^	IIn ^4^	Ain ^5^	GRAVY ^6^
				Hyd	Ac	Bas	Neu					
**A**	**Binase**	12.2	109	38.5	10.1	14.7	36.7	9.4	+3.90	27	79	−0.42
	**B1–20**	2.3	20	50.0	10.0	15.0	25.0	8.5	+0.79	36	117	0.14
	**B21–50**	3.1	30	43.3	6.7	10.0	40.0	8.4	+0.83	15	88	−0.14
	**B51–73**	2.5	23	30.4	13.0	17.4	39.1	9.7	+0.77	25	38	−1.08
	**B74–94**	2.5	21	38.1	14.3	9.5	38.1	4.4	−1.17	19	88	−0.37
	**B95–109**	1.9	15	26.7	6.7	26.7	40.0	9.5	+1.86	63	59	−0.76
**B**	**EGF**	6.2	53	30.2	17.0	13.2	40.0	4.8	−4.45	51	72	−0.43
	**TGFA**	5.6	50	36.0	12.0	16.0	36.0	5.93	−3.02	20	60	−0.08
	**HBEGF**	9.7	86	29.1	12.8	27.9	30.2	8.98	+8.07	49	70	−0.91
	**AREG**	10.1	87	20.7	12.6	27.6	39.1	9.35	+10.34	36	32	−1.54
	**EREG**	5.3	46	28.3	8.7	10.9	52.2	607	−1.35	8	68	0.06
	**EPGN**	14.7	132	38.6	9.1	13.6	38.6	7.32	+0.89	33	91	0.02
	**BTC**	9.0	80	25.0	12.5	17.5	45.0	7.65	+1.66	51	44	−0.79

^1^ length, number of amino acids (aa); ^2^ content of hydrophobic (Hyd), acidic (Ac), basic (Bas), and neutral amino acids in peptide; ^3^ net charge (NCh); ^4^ instability index (IIn); ^5^ aliphatic index (AIn); ^6^ grand average of hydropathicity (GRAVY).

**Table 2:** Prediction of anticancer peptides (ACPs) properties of binase-derived peptides as compared to known anticancer peptides. Scores above the threshold are in bold.

	Peptide	ACP ^1^	CPP ^2^	TP ^3^	HPho ^4^	APath ^5^	BI ^6^, kcal/mol
**A**	**B1–20**	0.63	−0.28	−0.80	−0.10	0.43	1.35
	**B21–50**	**0.73**	−0.53	−1.53	−0.07	0.45	0.84
	**B51–73**	0.47	−0.46	−0.60	−0.33	0.54	**3.46**
	**B74–94**	**0.73**	−0.56	−0.94	−0.16	0.23	**2.32**
	**B95–109**	0.69	−0.31	−0.89	−0.27	0.67	**2.98**
**B**	**LL–37**	**0.75**	−0.2	−1.58	−0.34	1.06	**3.00**
	**NRC–07**	**0.8**	−0.09	−0.9	−0.14	0.95	0.87
	**Roseltide rT7**	**0.72**	−0.47	**1.49**	−0.03	0.51	0.5
	**LfcinB**	**0.8**	0.18	−0.92	−0.34	0.98	**2.75**
	**HNp–1**	**0.83**	−0.13	**0.18**	−0.10	0.41	1.08

^1^ anticancer properties (ACP), ^2^ cell-penetrating properties (CPP), ^3^ toxic properties (TP), ^4^ hydrophobicity (HPho), ^5^ amphipathicity (APath), ^6^ Boman index (BI).

## Data Availability

The data presented in this study are available on request from the corresponding author.
